# Using social media for patient and public involvement and engagement in health research: The process and impact of a closed Facebook group

**DOI:** 10.1111/hex.13515

**Published:** 2022-09-22

**Authors:** Sophia Fedorowicz, Victoria Riley, Lisa Cowap, Naomi J. Ellis, Ruth Chambers, Sarah Grogan, Diane Crone, Elizabeth Cottrell, David Clark‐Carter, Lesley Roberts, Christopher J. Gidlow

**Affiliations:** ^1^ The Centre for Health and Development Staffordshire University Stoke‐on‐Trent UK; ^2^ Technology Enabled Care Programme, Staffordshire Sustainability and Transformation Partnership's Digital Workstream Stoke‐on‐Trent Clinical Commissioning Group Stoke‐on‐Trent UK; ^3^ Department of Psychology Manchester Metropolitan University Manchester UK; ^4^ School of Sport and Health Sciences Cardiff Metropolitan University, Cyncoed Campus Cardiff UK; ^5^ School of Medicine Keele University Keele Newcastle‐under‐Lyme UK; ^6^ Member Governor of Midlands Partnership NHS Foundation Trust Patient Representative Stoke‐on‐Trent UK

**Keywords:** patients, patient and public involvement, primary care, risk communication, social media, video‐stimulated recall

## Abstract

**Background:**

As part of a multifaceted approach to patient and public involvement and engagement (PPIE), alongside traditional methods, a closed Facebook group was established to facilitate PPIE feedback on various aspects of a project that used video‐recording to examine risk communication in NHS Health Checks between June 2017 and July 2019.

**Objective:**

To explore the process and impact of conducting PPIE through a closed Facebook group and to identify the associated benefits and challenges.

**Methods:**

Supported by reflections and information from project meetings used to document how this engagement informed the project, we describe the creation and maintenance of the Facebook Group and how feedback from the group members was obtained. Facebook data were used to investigate levels and types of engagement in the closed Facebook group. We reflect on the challenges of using this method of engaging the public in health research.

**Results:**

A total of 289 people joined the ‘Risk Communication of Cardiovascular disease in NHS Health Checks’ PPIE closed Facebook group. They provided feedback, which was used to inform aspects of the study, including participant‐facing documents, recruitment, camera position and how the methodology being used (video‐recorded Health Checks and follow‐up interviews) would be received by the public.

**Discussion:**

Using a closed Facebook group to facilitate PPIE offered a flexible approach for both researchers and participants, enabled a more inclusive method to PPIE (compared with traditional methods) and allowed rapid feedback. Challenges included maintaining the group, which was more labour intensive than anticipated and managing members' expectations. Suggestions for best practice include clear communication about the purpose of the group, assigning a group co‐ordinator to be the main point of contact for the group, and a research team who can dedicate the time necessary to maintain the group.

**Conclusion:**

The use of a closed Facebook group can facilitate effective PPIE. Its flexibility can be beneficial for researchers, patients and public who wish to engage in the research process. Dedicated time for sustained group engagement is important.

**Patient or Public Contribution:**

Patient representatives were engaged with the development of the research described in this paper and a patient representative reviewed the manuscript.

## 1 INTRODUCTION

Patient and public involvement and engagement (PPIE) is an integral part of health service development and delivery.^1^ The core concept of PPIE is that research is carried out ‘with’ or ‘by’ members of the public instead of ‘about’ or ‘for’ them.^2^ National Institute for Health Research (NIHR) states members of the public in the context of patient and public involvement include patients, potential patients, carers and people who use health and social care services as well as people from specific communities and from organizations that represent people who use services, people with lived experience of a health condition whether they are current patients or not.^3^ Increasingly funders, policy makers and research organizations in the United Kingdom expect health research to be carried out involving patients and members of the public. For example, for NIHR applications, it is compulsory to a have budgeted and resourced PPIE lead role and Wellcome and UKRI are also committed to PPIE. This involvement increases the likelihood of the needs of patients being met and results in more responsive services and improved health outcomes.^4^, ^5^

Patient and public involvement has been used in cancer research, exploring patient priorities for palliative care and identifying research priorities for cancer patients in treatment centres in the United Kingdom. Activities include the definition and prioritization of research topics, development of recruitment strategies, participating in data analysis and supporting dissemination.^6^ Patient and public involvement has been used in consultation activities and priority settings in health technology assessment such as projects summarizing the evidence on clinical effectiveness and safety of wearable cardioverter‐defibrillator therapy for primary and secondary prevention of sudden cardiac arrest in patients at risk and exploring patients' perspective regarding cervical cancer screening with human papillomavirus cotesting.^7^ Community members of the public and community‐engaged research patients who had experienced myocardial infarction were invited to take part in a focus group discussion to share past experiences and provide input and advice on the design of a research proposal for designing a clinical pharmacy primary care intervention for myocardial infarction.^8^

Challenges of carrying out meaningful PPIE include the time that needs to be dedicated to PPIE activities to accomplish the goals of the study and managing differing expectations of members of the research team.^9^ The positive impacts of working with the patient and public representatives are wide‐ranging and include developing user‐friendly research objectives, user‐friendly information and appropriate recruitment strategies resulting in enhanced quality of research.^10^ Members of a PPIE group exploring the experiences of people living with or caring for someone living with a mental illness have described the process as meaningful, enabling them to use their experiences to act as advocates for their community and helping them to reframe their narratives as ‘experts by experience’ following periods of acute illness.^1^ Having experience of a health condition often prompts individuals to seek knowledge of aetiology, prognosis and service provision, resulting in the knowledge that is both clinical and experiential.^11^ This knowledge positions the patient as an expert by experience, which can be complementary to traditional knowledge structures in healthcare in which the clinician is the sole source of expertize.^11^ By acknowledging this additional source of expertize, PPIE in health research positions patients and members of the public as actors undertaking or contributing to research, rather than simply as its recipients or beneficiaries.^12^ Various guidelines have been developed for traditional PPIE methods. The NIHR published standards for PPIE in health research^13^ and INVOLVE published their Values and Principle's Framework for best practice in PPIE.^12^ Commonly PPIE in health research takes place in highly regulated systems and environments, within stringent timelines.^14^ Traditional approaches to PPIE in healthcare include consultations, PPIE in health‐specific interest groups, lay membership on trust boards and PPIE committees, such as ‘Patient Participation Groups’ (PPGs).^4^ Relying on these methods can limit patient/public representation and statutory bodies controlling the nature and level of PPIE, contrary to the core concept of carrying out research ‘with’ patients and members of the public.^4^, ^12^ Recognizing these limitations, the project described in this paper, in addition to working with PPGs and healthcare professional representatives, utilized social media to set up a PPIE group to advise on the research.

Despite the aforementioned guidance on PPIE, guidance for using specific social media platforms is not comprehensive because different research approaches have different PPIE needs.^15^, ^16^ The needs of a study in developing a PPIE strategy can include but are not limited to a need to assess and understand the local context of the proposed study, a need to plan ahead and anticipate future issues, and a need to diversify and ethically support inclusive practice.^17^ As part of PPIE strategies to address these needs, members of the public can be involved in many different activities throughout the research cycle.^17^ For example, informing research priorities, informing design, ensuring methods are appropriate for the population under study, reviewing and commenting on participant facing literature, defining outcome measures, interpreting data, informing analysis, distributing findings, collaborating on reports, producing summaries and engaging in monitoring and evaluation processes.^17^

It has been established that a ‘one‐size fits all’ approach to PPIE is ineffectual, supporting the need for strategies tailored to each study.^18^

Reports of using social media to engage the public in research describe advantages, such as creating an established community with active engagement and knowledge exchange, and being able to include a larger, more diverse group of people than traditional face‐to‐face methods.^19^, ^20^ Using a closed Facebook group to facilitate PPIE is an accessible and cost‐effective method, and as Facebook is a platform familiar to many patients and researchers, no training is needed regarding its use.^21^ Facebook has been used to support user‐led research, cultivating collaboration between the public and experts,^22^ resulting in peer‐reviewed publications and producing evidence for a cross‐party parliamentary group.^22^

A challenge of using social media to support PPIE in research is the risk of excluding those who do not engage with social media, making it an appropriate adjunct to other PPIE methods.^15^

### 1.1 The current paper

This paper discusses the use of a closed Facebook group to facilitate PPIE for the RIsk COmmunication in NHS Health Check (RICO) study.^23^ PPIE was particularly prominent within RICO as it involved video‐recording primary care consultations and follow‐up video‐stimulated recall (VSR) interviews. The researchers recognized the need for sensitivity in the approach to ensure adequate recruitment, methods acceptability and participant experience. The current paper adds to the landscape of PPIE using social media literature by detailing the process and impact of using Facebook to facilitate PPIE, reporting on levels and types of engagement, how this informed the project and by providing a road map for navigating the challenges of using this method of PPIE to support health research.

### 1.2 Patient and public involvement in RICO

A proactive and multifaceted approach to PPIE was key in the development of the protocol for the RICO study, which explored how healthcare practitioners communicated CVD risk and how patients understood their risk during NHS Health Checks (NHSHC; Box 1 and 2).^24^, ^25^, ^26^

BOX 1 **NHS health checks**

NHS Health Check is a national prevention programme, which aims to reduce the chance of a heart attack, stroke or developing some forms of dementia. All eligible people aged 40–74 years should be invited for a Health Check, which screens for cardiovascular disease risk by assessing the top seven risk factors and providing individuals with behavioural support and, where appropriate, pharmacological treatment.

BOX 2 **RIsk COmmunication in NHS Health Check Project**

The RIsk COmmunication in NHS Health Check (RICO) project was a qualitative study with a quantitative process evaluation involving 12 general practices in the West Midlands of England. Practices were randomized to one of two groups: usual practice, in which practitioners used QRISK® 2 to assess and communicate cardiovascular disease (CVD) risk; and intervention, in which practitioners used JBS3 to assess and communicate CVD risk. In total, 173 Health Checks were video‐recorded and post‐Health Check, video‐stimulated recall interviews were conducted with 40 patients and 15 practitioners, using video excerpts to enhance participant recall and reflection. Risk communication, patient response and intentions for health‐protective behaviours were explored.

#### 1.2.1 Patient participation groups

PPGs are set up to be ‘critical friends’ of general practices. Since April 2015 every General Practice surgery is required to have a PPG to advise from the patient perspective in several different ways, to improve the effectiveness and quality of primary care services, and to support research. As part of the PPIE strategy for RICO, the research team attended meetings with three different PPGs to determine several aspects of the project protocol.

Forty‐eight patients and six practice staff attended four PPG meetings between January and April 2016 across three Staffordshire practices. Discussions focussed on the perceived importance and necessity of research into risk communication in NHSHCs, and the acceptability of specific methods that the project would use to study complex practitioner–patient interactions around CVD risk. Much discussion was around the acceptability of video‐recording consultations. The research team also sought feedback from the group regarding procedures, such as obtaining consent, VSR protocols, interview duration and opinions about methods to engage patients in PPIE activities throughout the study. These activities included being part of the study steering group, taking part in mock NHSHCs, commenting on participant‐facing study information, and using a closed Facebook group as a form of virtual PPIE. Eighteen patients and one member of the practice staff volunteered to contribute to these activities: seven offered to sit on the project steering committee, four offered to join the closed Facebook group, 11 offered to take part in a mock NHSCH and 10 offered to comment on participant facing study information.

#### 1.2.2 Steering group

The project steering group met four times during the study, with membership including an independent expert chair, two independent academics, a practice nurse who delivered training in NHSHCs, two public/patient representatives and members of the study team. Having patient steering group members is a common way to ensure their input into project management and involve them in major decisions for the study (e.g., protocol amendments, extension requests). Steering group members received a progress update at each meeting whereby their thoughts and ideas were retrieved regarding challenges experienced by the research team.

#### 1.2.3 Mock NHSHCs

Four patients and one Healthcare Assistant, from one practice, took part in mock NHSHCs to test the protocols and practicalities of the proposed methods. The mock NHSHCs informed how data collection would run in practice, the time required to set up the recording equipment, what was needed to ensure consistent set up of the recording equipment in different environments, meeting patients' preconsultation to allow for verbal explanation and consent, debriefing the participant post‐NHSHC and the appropriate protocol for separating out audio and video files for transcription, alongside developing protocols for VSR interviews (including the interview schedule).

#### 1.2.4 The closed Facebook group

PPIE contributors are often required to be a representative of an entire population, while holding both the status of a ‘lay person’ and the skills and knowledge necessary to engage with professionals on their terms.^4^ This, combined with a reliance on self‐selection or the purposeful selection of acquiescent or socioeconomically advantaged individuals, results in a select few voices being heard in PPIE fora and the underrepresentation of marginalized groups.^11^

Using Facebook to facilitate a PPIE group was discussed at two PPG meetings and welcomed as a means of reaching patients that span the NHSHC age range (40–74 years) and to accommodate different preferences for communication. Facebook is one of the most widely used platforms in the United Kingdom, used by 73% of people aged 16–64 years.^27^ In addition to traditional means (engaging with PPGs and public members of the Steering Group), a closed Facebook group was appropriate for several reasons. First, it supported ongoing PPIE throughout the project, providing a means of receiving feedback from people representing the patient group on participant‐facing materials, procedures for obtaining consent within practices, the organization of VSR interviews, reports to disseminate findings to participants and patient groups and recommendations for practice. Second, the study team recognized the need for quick engagement with, and feedback from, patients and the public to develop acceptable protocols and address issues as they arose. Third, NHSHC is a national programme and social media is a cost‐neutral means of enabling a wider, more diverse group of people to be involved compared with traditional methods.

## 2 METHODS

### 2.1 Building and maintaining the closed Facebook group

**Figure 1:**
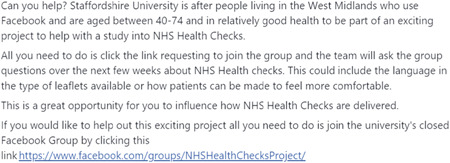
Recruitment advertisement posted in General Practice Facebook groups.

A closed Facebook group named ‘Risk Communication of Cardiovascular disease in NHS Health Checks’ was created. The research team worked with Redmoor Health, an organization that supports the management of general practice Facebook groups. Redmoor initially set up the closed Facebook group and facilitated recruitment to the closed Facebook group by posting advertisements to General Practice Facebook groups (Figure 1). The research team took over the sole management of the group after 2 months.

Recruitment was targeted at the Facebook pages of general practices in the Stoke‐on‐Trent, Staffordshire area, with a 35‐mile radius and within the age group that people qualify for an NHSHC (40–74 years). The recruitment post was published on 31 Facebook pages and the average reach for each post was 451, meaning that about 451 people came across each recruitment post on their Facebook feed. Some of the more engaging Facebook pages had a higher impact (e.g., for one General Practice Facebook page, the invitation reached 1599 individuals).

**Figure 2:**
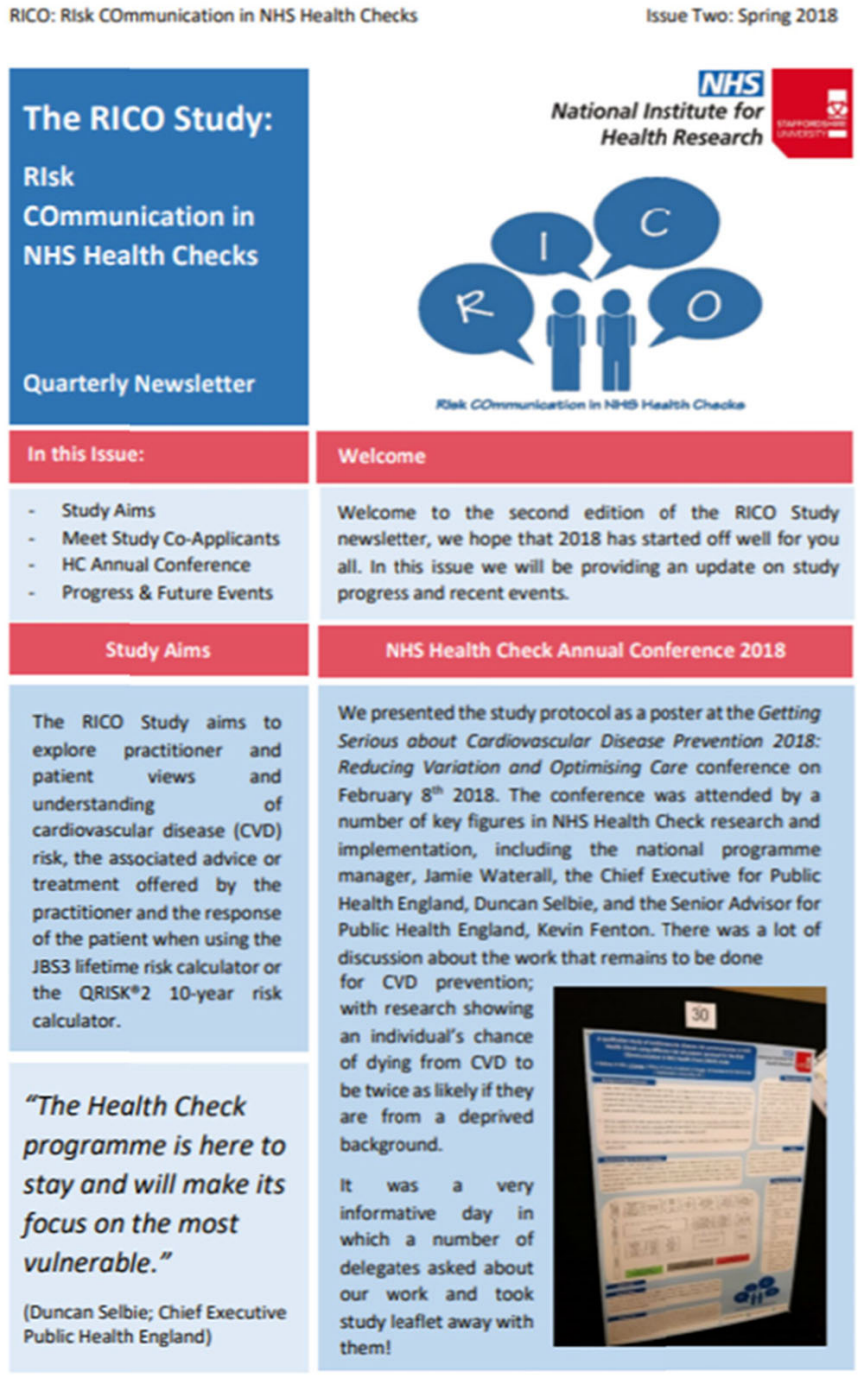
RIsk COmmunication in NHS Health Check (RICO) newsletter.

Between June 2017 and July 2019, the research team used four types of posts when engaging with the PPIE group: posts disseminating the RICO newsletter, question posts, information posts and polls. Within each type, some posts focused on the project and some focused on encouraging discussion or providing information to the group that was not specifically related to the project, although always related to the topic of heart health (and, therefore, relevant to RICO). The research team authored a newsletter about the RICO project to keep the PPIE group updated, which was distributed quarterly (see Figure 2). This newsletter was shared directly in the closed Facebook group, with the steering group and publicly on social media. Five editions of the newsletter were distributed over the course of the project and gave introductions to the study team, detailed up‐to‐date progress on the project, next steps and reported attending conferences to disseminate findings from the project.

### 2.2 Gaining feedback from the group

**Figure 3:**
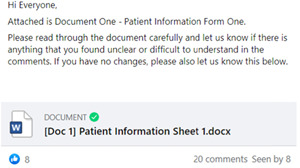
Facebook group post requesting feedback from patient and public involvement and engagement group members.

Members of the group reviewed all participant‐facing documents, including patient participant information sheets and consent forms (Figure 3). Posts asking for feedback on these documents clearly stated what was required (e.g., for members to view the document, state if they found anything difficult to understand and comment on the post with their feedback). These posts had a Word document attached, which the group members could download or view online.

**Figure 4:**
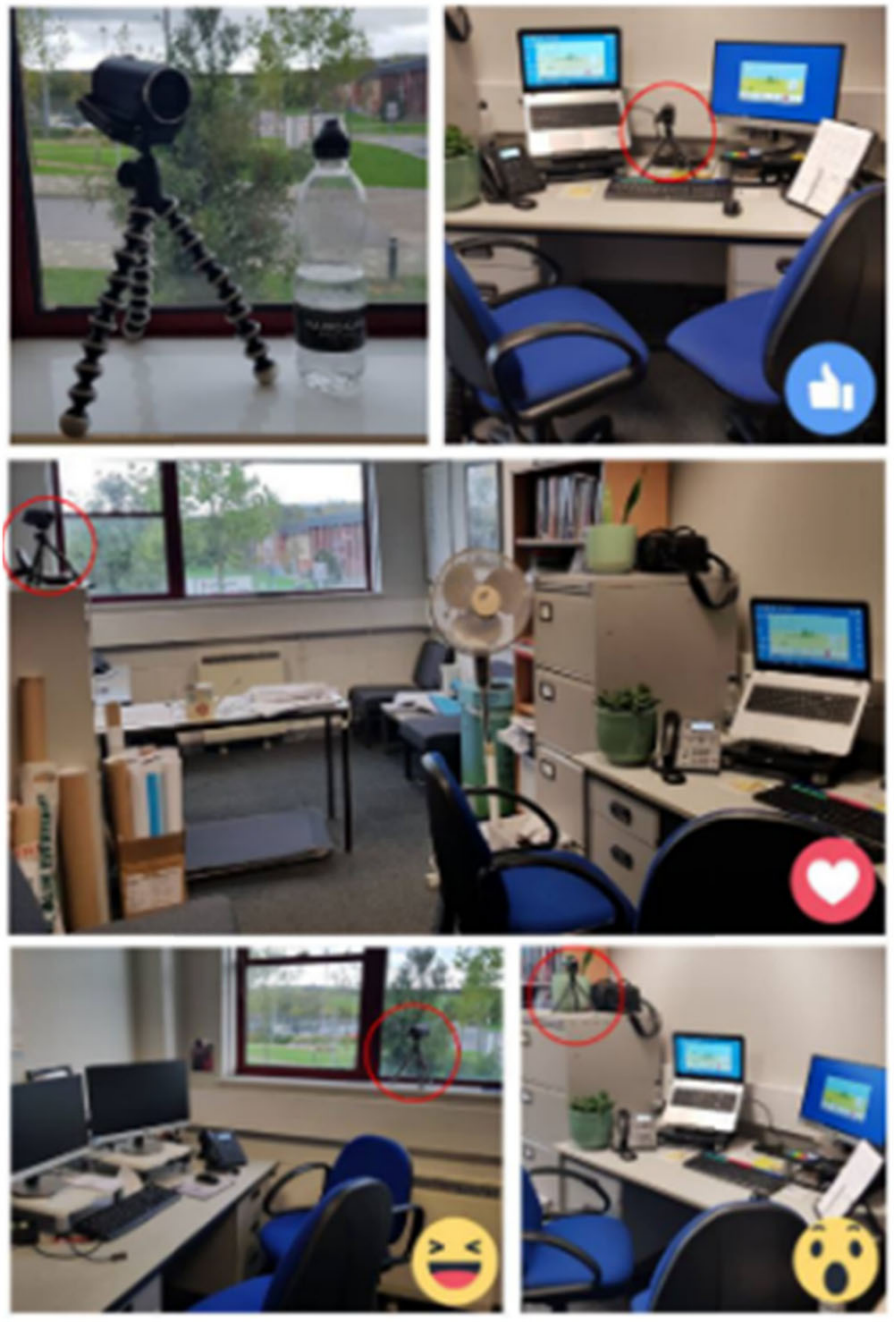
Facebook group post requesting opinions about camera position.

**Figure 5:**
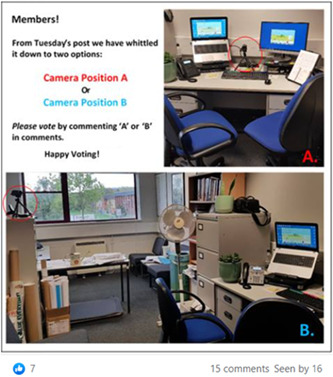
Facebook post requesting opinion about camera position.

Members of the PPIE group were consulted about the most appropriate place to put the camera in the consultation room for the video‐recorded NHSHCs (Figures 4 and 5). Two Facebook posts created by a member of the research team provided images of possible positions in the consultation room where a camera could be placed to record the NHSHC for the study, alongside a picture of the camera itself with a bottle of water for size comparison. Members of the group were asked to indicate their preferred camera position using the like, love, laugh or wow reaction icons on Facebook.

### 2.3 Facebook data

Information provided by Facebook was used to explore the process and impact of the closed Facebook group as a method of PPIE. Group members provided demographics when they joined the group, including their age, gender and location. The research team was also able to see engagement levels at different times across each day of the week, and how much group members engaged with different types of posts (question, information and poll). How the group members chose to engage could also be seen by how many ‘likes’ or comments each post received and how many votes were cast in each poll post. The data are explored using descriptive statistics.

As this paper discusses the use of a closed Facebook group to facilitate PPIE for the RICO study no ethical approvals were sought.

## 3 RESULTS

### 3.1 Group member demographics

The PPIE closed Facebook group had a total of 289 members (five of these provided no information about their age or gender; Table 1). Most were from Staffordshire in England (*n* = 261), with small numbers from Greater Manchester (*n* = 3), Birmingham (*n* = 2), Cheshire (*n* = 4) and one each from Lancashire, Surrey, Merseyside, Shropshire, Leicestershire, Cumbria, South Yorkshire and Berkshire. There were also a small number from outside England: India (*n* = 3), Canada (*n* = 1), Iraq (*n* = 1), the Philippines (*n* = 1) and Pakistan (*n* = 1). Although individuals who were eligible for an NHSHC were preferred (as stated in the recruitment advertisement, Figure 1), Redmoor Health and the research team did not enforce these criteria when admitting individuals into the closed Facebook group.

**Table 1:** Demographics of PPIE group members

Age range (years)	Women		Men		Total	
	*n*	%	*n*	%	*n*	%
18–24	3	1.1	2	0.7	5	1.7
25–34	4	1.4	2	0.7	6	2.1
35–44	39	13.7	8	2.8	47	16.5
45–54	88	31.0	17	6.0	105	36.9
55–64	68	23.9	3	1.1	71	25.0
65+	42	14.8	8	2.8	50	17.6
Total	244	85.9	40	14.0	284	

Abbreviation: PPIE, patient and public involvement and engagement.

### 3.2 Popular days and times for engagement

For clarity, engagement in this context refers to a member of the group liking or commenting on a post. Mondays, Tuesdays and Fridays were the most popular days of the week for engagement, with the least engagement observed on Thursdays. The most popular times of the day for engagement were 19:00 (an average of 13 engagements) and 20:00 (an average of 10 engagements). The hours between 02:00 and 05:00 were least popular (0 engagements), 00:00, 01:00, 06:00, 09:00, 12:00 and 23:00 (1 engagement).

### 3.3 Overall engagement with posts

In total, researchers posted 30 question posts (19 project‐related; 11 nonproject‐related), 32 information posts (11 project‐related; 21 nonproject‐related) and 9 polls (5 project‐related; 4 nonproject‐related). Overall engagement with posts totalled 437 likes and 352 comments. People commented more often on posts related to project development compared with those unrelated to project development (Table 2).

**Table 2:** Number of likes, comments and people who commented for three categories of posts used in the closed Facebook group

	Question posts						Information posts						Poll posts					
	Project development posts		Nonproject development posts		Total		Project development posts		Nonproject development posts		Total		Project development posts		Nonproject development posts		Total	
	*n*	% Of question posttotal	*n*	% Of question posttotal	*n*	% Of question posttotal	*n*	% Of information posttotal	*n*	% Of information posttotal	*n*	% Of information posttotal	*n*	% Of poll posttotal	*n*	% Of poll posttotal	*n*	% Of poll posttotal
Total posts	19	63.3	11	36.6	30	100	11	34.3	21	65.6	32	100	5	55.5	4	44.4	9	100
Posts that used an image	11	52.3	10	47.6	21	100	7	29.16	17	70.8	24	100	0	0	0	0	0	–
Number of likes received	98	73.6	35	26.3	133	100	127	52.9	113	47.0	240	100	38	59.3	26	40.6	64	100
Number of comments received	202	77.3	59	22.6	261	100	36	78.2	10	21.7	46	100	34	75.5	11	24.4	45	100
Number of people who commented on a post	186	77.8	53	22.1	–	–	32	76.1	10	23.8	–	–	34	75.5	11	24.4	–	–

Poll posts elicited the fewest likes (*n* = 64) and comments (*n* = 45), which is expected given their primary purpose was to encourage people to vote for one of the proposed options. Five polls related to project development received an average of 23.6 votes per poll (range: 15–32) and approximately 76% of the comments left on poll posts (*n* = 34; total *n* = 45). Information posts received the most likes (total *n* = 240); over half of these posts were project‐related (*n* = 127), despite the project‐related posts accounting for approximately one‐third of the total (*n* = 11). Question posts received 133 likes (project posts *n* = 98; nonproject posts received *n* = 35), and 261 comments (project posts *n* = 202; nonproject posts *n* = 59). Overall, more people commented on the project development posts (*n* = 186) compared with nonproject development posts (*n* = 53). This was expected as many of the questions asked for feedback on documents and opinions based on elements of recruitment and study design. All patterns observed are based on descriptive statistics and should be interpreted accordingly.

### 3.4 Impact of the group's feedback

Members of the group were asked to feedback on several aspects of the study. The group provided feedback on seven participant‐facing documents and an average of six people commented on each document. The first post requesting feedback on where to place the camera to record NHSHCs (Figure 3) received five ‘like’ and four ‘love’ reactions, indicating that two positions were favoured. To follow up, an additional post was made providing a choice between camera positions A and B and asking members to indicate their preferred camera position in the comments (Figure 4). This post received 15 comments, all but one indicating they would prefer position B. The group also provided valuable feedback on the recruitment processes at several time points and shed light on how it may feel for participants re‐watching clips of their NHSHC during the VSR interview.

PPIE group member engagement impacted the RICO study in several ways. First, feedback about participant‐facing documents, such as consent forms and information sheets, for patient and practitioner participants. PPIE group members raised concerns about user‐friendly language, ensuring that the information sheet was particularly clear about the methods to avoid confusion. This resulted in greater clarity regarding the method being used and that all patients and practitioners who took part knew which parts would be video‐recorded.

Second, feedback about camera placement made it clear that the camera should be placed out of view of the participant, subject to what the consultation room would allow. Consultation rooms in practices varied in size and shape, but the researchers were able to adhere to the group's preference and it was effective in capturing the consultations without disturbing the natural flow of the NHSHC.

Third, given the slower than anticipated recruitment, the group was asked for input on several aspects related to this. Poor response to initial mail‐outs from practices inviting patients to take part in the study led the research team to seek group feedback on reasons and possible solutions. For one practice, the group advised that the response timeframe was too short and potentially off‐putting, as people needed more time to think about what is being asked of them, and to fit it into their lives. The research team fed this back to the practice, who in turn changed their recruitment letter to allow people more time, and recruitment improved greatly.

## 4 DISCUSSION

This paper describes the use of a closed PPIE Facebook group for rapid engagement with and feedback from patients and the public regarding a primary care‐based research project. A total of 289 people joined the closed PPIE Facebook group for the RICO study. Members of this group provided their opinions and feedback on many aspects of the project, most notably the participant‐facing documents, camera position, how the video‐recording of consultations would be received and the recruitment process.

### 4.1 Benefits of using a Facebook group to facilitate PPIE

Using one of the most widely used and free to access social media platforms resulted in the PPIE Facebook group being potentially more inclusive than PPIE groups recruited through traditional methods in healthcare research (e.g., through PPGs). A central issue with PPGs and other PPIE reference groups is the unrepresentative membership of the wider patient base, specifically over‐representing people who are white, middle class, retired or semi‐retired.^4^, ^28^ This is partially the result of structurally unequal selection processes leading to public participation initiatives that represent some subgroups more than others.^11^, ^28^ The research team recognizes this limitation and holds the position that ‘expertize’ is not limited to certifiable qualifications, but also includes expertize acquired through experience.^29^ Using a closed Facebook group to facilitate PPIE enabled researchers to increase the size and diversity of the pool of potential PPIE participants.^20^ A total of 289 members, across age groups and from different areas of the United Kingdom, were able to join the group and see what the research team was doing and provide feedback. The research team created a newsletter for the group detailing the development of the project to support transparency, alongside asking questions about different aspects of the project. Subsequently, 289 members of the public were able to oversee and contribute to the project between June 2017 and July 2019.

Members did not need to travel to attend meetings, which enabled people who would be excluded from traditional face‐to‐face PPIE meetings due to mobility or financial barriers, as many PPIE approaches ask that the person spend money on travel and then claim it back as expenses. Additionally, members of the group engaged at a time or day that is convenient for them. This means that people in full‐time employment, who have caring responsibilities or generally struggle to find whole afternoons or mornings to attend workshops or long meetings are able to be involved.

Further, the PPIE Facebook group allowed for a more flexible and less intensive kind of PPIE. Using a Facebook group allowed the researchers to seek feedback in a variety of ways from the group, such as comments, likes and voting in polls. Letting people vote with polls or by using the like button lets them have their say without having to engage in big discussions if they do not want to. People can leave comments on posts to detail their thoughts and opinions on different areas or they can leave one‐word responses. These benefits have the potential to open PPIE up to a different cohort of people who previously would have found it difficult to commit to being a member of a PPG, a series of workshops or stakeholder events are often the chosen method of including members of the public in research. This potential is evident by the large number of people successfully recruited to Facebook who are generally younger than expected (compared with traditional PPGs).

As Facebook has been active as a social networking platform since 2004, and it is one of the most popular platforms in the United Kingdom, it is widely used.^27^ This meant that the research team and the people who joined the closed PPIE group already had a working knowledge of the platform as they already had established profiles. A benefit of this is that it was not necessary to train the researchers or PPIE group members on how to use Facebook before the project started, which saved time.^21^

### 4.2 Challenges of using a Facebook group to facilitate PPIE

Maintaining the Facebook group was a challenge for researchers. It required considerably more time than anticipated to keep up‐to‐date with posts and replies. This was particularly true at the beginning as new members joined the group, often with questions or comments that required a reply, or simply ensuring that all members were welcomed. As detailed above the group members engaged most in the evenings, meaning that the responsible researcher engaged in discussion with group members outside usual working hours. This resource issue was exacerbated as the project overran the original completion date and staffing was reduced. As a result, ongoing group engagement was not adequately maintained and opportunities for dissemination and possible engagement of group members in future research were missed.

Some group members appeared unclear about the purpose of the Facebook group. For example, some members sought medical advice from the researcher about their personal cardiovascular health, even contacting the researcher privately through their personal Facebook account to pursue medical advice after their initial enquiry was not considered to have been answered satisfactorily. Consequently, the research team regularly stated that they were not medically trained and urged individuals to contact their GP if they have concerns about their health.

In addition to these challenges, a limitation of this approach is the exclusion of those without reliable internet access or who are unfamiliar with social media. However, alongside traditional PPIE methods, these limitations were somewhat mitigated.

Finally, the decision not to enforce specific criteria for members of the closed Facebook group (e.g., eligibility for an NHSHC) could have affected the feedback received. It was clear from several comments that some group members were living with cardiovascular health issues, which would preclude them from NHSHC. As discussed previously, people with experience of specific health conditions develop a knowledge and understanding of that condition, which would not be expected of the target population for NHSHC, for whom CVD prevention is the programme target.

### 4.3 Suggestions for best practice

Reflecting on this experience, suggestions for those seeking to pursue a similar approach include:

*Sufficient resources*. To facilitate the group effectively and dedicate the necessary attention, a group co‐ordinator should be appointed, with responsibility for providing clarity of the purpose of the group, creating and monitoring posts and associated activity, engaging with members and managing expectations.
*Sustainability*. Ensure support beyond the life of the project, when dissemination continues, and follow‐on studies may be developed. The continuity of PPIE group members throughout could be of great benefit and continued engagement with the group would facilitate easier access to future feedback, if required.
*Boundaries*. Set boundaries by ensuring that all members know the role of the research team and that this cannot extend to medical advice.
*Flexible working*. It is important that the group is monitored at specific times and days, for example, 9:00–17:00 on weekdays, but also with availability outside working hours. This can facilitate smooth communications and prevent confusion.
*Timing of posts*. PPIE group engagement was highest between the hours of 19:00 and 20:00 and posts asking for feedback on specific questions elicited the most likes and comments (e.g., camera position). Therefore, our recommendation is that posts be scheduled in the evening and, if possible, a member of the research team be available to respond to comments from group members.

## 5 CONCLUSION

Facebook can provide a platform for successful PPIE in health research with several advantages, particularly when used alongside traditional methods. Using a closed Facebook group to facilitate PPIE offers flexibility that can be beneficial for both research teams and patients and the public who wish to engage in the research process. This approach allows for wider participation, and in the main requires no training.

## AUTHOR CONTRIBUTIONS

*Conceptualization, funding acquisition and methodology*: Christopher J. Gidlow, Naomi J. Ellis, Ruth Chambers, Sarah Grogan, Diane Crone, Elizabeth Cottrell, David Clark‐Carter. *Investigation*: Lisa Cowap, Victoria Riley, Naomi J. Ellis. *Supervision*: Christopher J. Gidlow, Naomi J. Ellis, Ruth Chambers, Sarah Grogan, Diane Crone, Elizabeth Cottrell. *Writing original draft*: Sophia Fedorowicz. *Writing reviewing and editing*: Sophia Fedorowicz, Victoria Riley, Sarah Grogan, Lisa Cowap, Christopher J. Gidlow, Ruth Chambers, Lesley Roberts.

## CONFLICTS OF INTEREST

The authors declare no conflicts of interest.

## Data Availability

Data sharing is not applicable to this article as no datasets were generated or analysed during the current study.
